# A159 OPIOID USE IS DECLINING AMONG PEOPLE WITH INFLAMMATORY BOWEL DISEASE: A POPULATION-BASED STUDY

**DOI:** 10.1093/jcag/gwab049.158

**Published:** 2022-02-21

**Authors:** E Kuenzig, J Mason, C N Bernstein, T Gomes, D Juurlink, G G Kaplan, J Peña-Sánchez, L E Targownik, S Vigod, J Begum, Z Nugent, E I Benchimol

**Affiliations:** 1 Gastroenterology, Hepatology and Nutrition, The Hospital for Sick Children, Department of Pediatrics, University of Toronto, Toronto, ON, Canada; 2 Centre for Addiction and Mental Health, Toronto, ON, Canada; 3 University of Manitoba, Winnipeg, MB, Canada; 4 University of Toronto, Toronto, ON, Canada; 5 Medicine and Community Health Sciences, University of Calgary, Calgary, AB, Canada; 6 Department of Community Health and Epidemiology, University of Saskatchewan, Saskatoon, SK, Canada; 7 Women’s College Hospital, Toronto, ON, Canada; 8 ICES, Toronto, ON, Canada

## Abstract

**Background:**

Patients with inflammatory bowel disease (IBD) are more likely to use opioids than those without IBD and are more susceptible to the negative consequences of opioid use, including increased risk of death.

**Aims:**

Examine trends in the use of opioids among people with and without IBD, where opioids were prescribed, and who prescribed them.

**Methods:**

We identified Ontarians with IBD (7/2012–3/2017) from population-based health administrative data using validated algorithms. We matched each patient with IBD on age and sex to 5 people without IBD. We calculated age- and sex-standardized quarterly rates of patients taking opioids, characterized as any, chronic (>90 days), or acute (≤90 days) among people with and without IBD. Among people with IBD, we identified the location of the last healthcare interaction prior to filling the prescription (hospital, emergency department [ED], outpatient clinic), presuming this was where the opioid was prescribed. Opioids prescribed after outpatient visits were stratified by specialty (gastroenterologist, family physician/internist, surgeon, other). Average quarterly percentage change was calculated using Poisson regression, adjusting for age, sex, income, and rural/urban household.

**Results:**

Of 92,233 IBD patients (mean 47 y at study entry, 45% male, 50% Crohn’s), 56% had at least 1 opioid prescription during the study period. Opioid use was more common among people with IBD (any: IRR 2.11, 95% CI 2.08–2.14; chronic: IRR 2.61, 95% CI 2.54–2.69; acute: IRR 1.82, 95% CI 1.79–1.94), Figure A. Among IBD patients, any opioid use decreased by 0.5% (95%CI 0.4–0.5) per quarter, from 13.4% (95%CI 13.1–13.6) to 12.7% (95%CI 12.5–13.0). Chronic opioid use decreased by 0.3% (95%CI 0.2–0.4) per quarter while acute opioid use among IBD patients decreased by 0.6% (95% CI 0.5–0.7) per quarter. Most opioids were prescribed after an outpatient visit (70.2%), most often (82.7%) by a family physician or internist. Total outpatient prescriptions declined by 1.9% (95%CI 1.7–2.0) per quarter, from 5.8% (95%CI 5.6–5.9) to 3.8% (95%CI 3.7–3.9). Relative quarterly decreases were similar across all specialties, Figure B.

**Conclusions:**

Opioid use remains common among IBD patients but has decreased since 2012. The prescriptions most often originated after outpatient visits to family physicians and internists rather than gastroenterologists or surgeons.

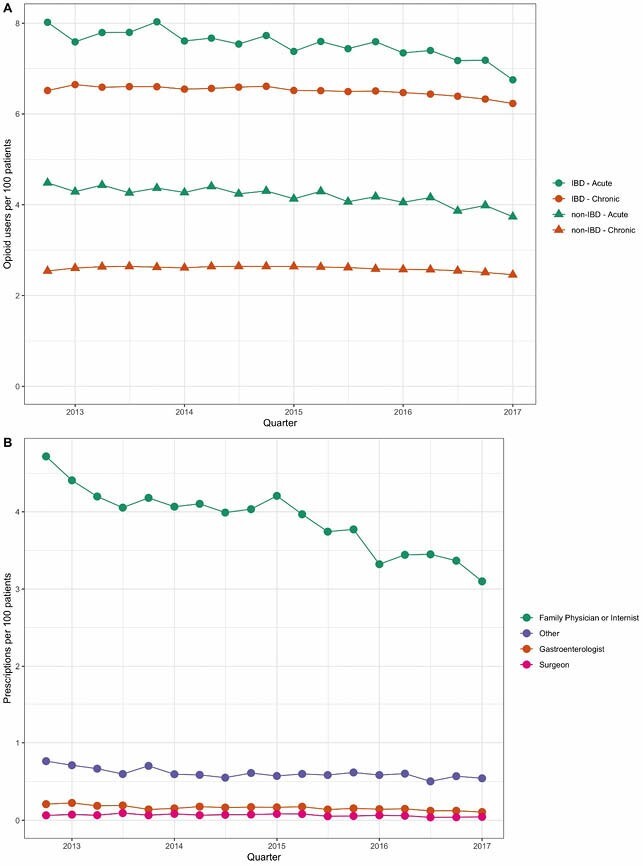

Trends in (A) the acute and chronic opioid use among those with and without IBD and (B) the specialist prescribing opioids to IBD patients when the most recent healthcare contact was an outpatient visit.

**Funding Agencies:**

American College of Gastroenterology

